# Notoginsenoside R1, a metabolite from Panax notoginseng (Burkill) F.H.Chen, stimulates insulin secretion through activation of phosphatidylinositol 3-kinase (PI3K)/Akt pathway

**DOI:** 10.3389/fphar.2024.1478917

**Published:** 2024-09-27

**Authors:** Altaf Al-Romaiyan, Ahmad Barakat, Sulaiman K. Marafie, Willias Masocha

**Affiliations:** ^1^ Department of Pharmacology and Therapeutics, College of Pharmacy, Kuwait University, Kuwait; ^2^ Biochemistry and Molecular Biology Department, Dasman Diabetes Institute, Kuwait

**Keywords:** mouse islets, Panax notoginseng, insulin secretion, plant extract, diabetes

## Abstract

**Background:**

For ages, botanical medicine has been used in the treatment of diabetes mellitus (DM). Notoginsenoside R1 (NGR1), a Panax notoginseng (Burkill) F.H.Chen metabolite, has been documented to possess antidiabetic action *in vivo*. However, its precise molecular mechanism of action is not clear.

**Objectives:**

We evaluated NGR1’s effects on blood glucose *in vivo* and then evaluated *in vitro* whether NGR1 has effects on insulin secretion and the probable molecular pathways involved in NGR1-induced insulin secretion.

**Methods:**

Diabetes was induced in mice by streptozotocin. Glucose tolerance test was performed before and after NGR1 was administered intraperitoneally to diabetic animals for 4 weeks. Static and perifusion experiments were performed using isolated female BALB/c mouse islets. Preproinsulin (*Ins*) mRNA expression was measured using q-PCR. Protein expression of PI3K/Akt pathway was assessed using the fully automated Wes™ capillary-based protein electrophoresis.

**Results:**

Treatment of diabetic mice with NGR1 improved their glucose intolerance. *In vitro*, NGR1 increased insulin secretion in a concentration-dependent manner. NGR1 initiated the secretion of insulin at 2 mM glucose and augmented glucose-stimulated insulin secretion which was sustained throughout NGR1 perifusion. NGR1-induced insulin secretion was not altered by a voltage gated calcium channel blocker or protein kinase A inhibitor. NGR1 did not significantly modulate *Ins* mRNA expression. However, NGR1 significantly increased the levels of phospho-Akt and phopho-p-85.

**Conclusion:**

In conclusion, this study has shown that NGR1 ameliorates hyperglycemia in diabetic mice. NGR1 has a direct insulin secretagogue activity on mouse islets, stimulates insulin secretion at both basal and postprandial glucose concentrations, and activates PI3K/Akt pathway to induce insulin secretion. These results suggest that NGR1 may provide an alternative therapy to manage DM.

## 1 Introduction

Approximately 537 million persons, between the ages 20 and 79 years, worldwide suffered from diabetes mellitus in 2021 (IDF, 2021). The introduction of new classes of drug in recent years has significantly transformed the pharmacotherapy of type 2 diabetes mellitus (T2DM). The goal is tight glycemic control, in order to lower the risk of microvascular and macrovascular complications and enhance the patient’s quality of life. Although new classes of medications have been introduced, adequate glycemic control remains below target for most T2DM patients. This may necessitate treatment with multiple drugs, which increases the risk of drug-drug interactions and adverse drug events. Therefore, an area of active research is identifying new and safe drugs, especially of plant origin, for the treatment of T2DM.

Panax notoginseng (Burkill) F.H.Chen (PNG) has been used traditionally as part of Chinese medicine for centuries (Ng, 2006). The plant belongs to the Araliaceae family. The medicinal effect of PNG resides in its roots, thus, PNG root extracts have been suggested as immunologic adjuvants (Scaglione et al., 1990; Sun et al., 2003), anti-inflammatory (Rui et al., 2009; Wang et al., 2011), hemostatic (White et al., 2000), anti-cancer (Wang et al., 2007), cardio-protective (Fan et al., 2012; Hong et al., 2009; Lau et al., 2009; Yuan et al., 2011; Zhang et al., 2008) and antidiabetic agents (Uzayisenga et al., 2014; Yang et al., 2010). The active metabolites that are believed to be responsible for the pharmacological activity of PNG are PNG saponins (PNS). There are 30 different PNS that have been isolated so far. Out of these 30 saponins, notoginsenoside R1 (NGR1) has been recently identified as one of the new and promising metabolites of PNG for treating various diseases.

Notoginsenoside R1 has been reported to have a wide range of beneficial effects against cancer (Cai et al., 2021; Li et al., 2022b; Qin et al., 2021), inflammation (Li and Huang, 2021; Sun et al., 2019), cardiovascular (Li et al., 2022a; Liu et al., 2021; Liu et al., 2019; Zeng et al., 2023; Zhu et al., 2021), neurological (Hu et al., 2020; Liu et al., 2022; Luo et al., 2022; Yang et al., 2024) and hepatological (Gong et al., 2022) diseases. It has also been suggested to have antidiabetic effects *in vivo*. Nevertheless, the data regarding its antidiabetic effect are conflicting and scarce. Some studies showed that NGR1 improved glucose tolerance (Huang et al., 2016; Zhai et al., 2018; Zhang et al., 2018) while others showed no improvement in glycemic control (Yang et al., 2010; Zhang et al., 2019) in animal models of diabetes *in vivo*. The discrepancy between these studies could be attributed to the differences in the design of the experiments and complexity of the animal models used. In some of the studies reporting glucose-lowering effects of NGR1, the exact mechanism by which NGR1 decreased blood glucose was not investigated and in some studies the mechanism was attributed to the amelioration of insulin resistance. These studies did not identify if NGR1 increased insulin secretion. Indeed, there are no studies that tested whether NGR1 has a direct effect on the function of pancreatic islets of Langerhans and insulin secretion. Using our new custom-designed screening method, reviewed in detail in (Al-Romaiyan et al., 2023b), we investigated whether NGR1 could have the potential to be a plant-derived pancreatic beta-cell directed antidiabetic agent.

In this study, we examined the effect of NGR1 on blood glucose levels in a streptozotocin (STZ)-animal model of diabetes *in vivo* as a proof-of-concept experiment. We then investigated whether NGR1 has direct effects on insulin secretion using isolated primary islets *in vitro*. There are important effectors that mediate the coupling of stimulus and secretion in pancreatic β-cells. Examples of these mediators are calcium, cyclic adenosine monophosphate (cAMP) and phosphatidylinositol 3-kinases (PI3K). These second messengers in turn activate protein kinases (PK) such as PKA and PKB, also known as Akt, respectively, which are involved in insulin exocytosis and release. Therefore, we also examined the expression of the total and phosphorylated forms of these molecules to find a possible signaling pathway that NGR1 might use in β-cells to increase insulin secretion *in vitro*.

## 2 Materials and methods

### 2.1 NGR1 preparation

NGR1 is a purified metabolite from the root of Panax notoginseng (Burkill) F.H.Chen. Panax notoginseng (Burkill) F.H.Chen is the accepted plant name of a species in the genus Panax (Family Araliaceae) and was validated taxonomically by http://mpns.kew.org/mpns-portal/. NGR1 was readily available to purchase from Sigma-Aldrich (United States). NGR1 (purity >98%, Sigma-Aldrich, United States) was dissolved in sterile H_2_O to produce a 1 mg/mL stock. The stock was stored at −20°C for further use and diluted in physiological buffer (Gey and Gey, 1936) or tissue culture media to the desired concentration on the day of the experiments for *in vitro* experiments or used as such for *in vivo* experiments.

### 2.2 Animals

Eight to twelve weeks old female BALB/c mice (25–30 g in weight) used in this study. Animals were maintained on a 12 h light/dark cycle and had free access to standard rodent food and water. All procedures were approved by Kuwait University’s Health Science Center (HSC) Ethical Committee for the Use of Laboratory Animals in Teaching and Research (29/VDR/EC/3737).

### 2.3 *In vivo* experiments

#### 2.3.1 *In vivo* drug treatment

Eight-weeks-old BALB/c female mice were left for 1 week in the experimental rooms for acclimatization. They were randomly divided into 3 different groups: 1) control (non-diabetic) + vehicle group, 2) STZ (diabetic) + vehicle group, and 3) STZ (diabetic) + NGR1 group. Diabetes was induced by STZ as detailed in the previously published protocol (Furman, 2015). Following a 4 h fast, a single high dose of STZ (200 mg/kg) in 0.1 M sodium citrate buffer (pH 4.5) was injected to animals Intraperitoneal (IP) injection. An equal volume of sodium citrate buffer (pH 4.5) was injected by IP to control animals. Repetitive injections of 200 mg/kg STZ were carried out if needed. Animals in the diabetic groups were included in the experiment if they were glucose intolerant following a glucose tolerance test (GTT). At week 0, NGR1 at a dose of 10 mg/kg/day or its vehicle (sterile water) was administered intraperitoneally to the animals. The animals were treated with NGR1 for 4 weeks. Various doses of NGR1 have been used previously by other researchers. The dose of NGR1 was selected based on previously published studies (Gui et al., 2014; Huang et al., 2016).

#### 2.3.2 Glucose tolerance test

Glucose tolerance test was carried out before and after NGR1 administration as previously described (Al-Romaiyan et al., 2013). Two g/Kg of glucose (20% solution) was injected intraperitoneally to mice after an overnight fast. Blood glucose levels before injection (0 min) and at 30, 60, 90 and 120 min after glucose injection were recorded using Accu-Check glucometer (Roche, United States).

### 2.4 Maintenance of cell lines

Rat insulinoma cells, INS-1 832/13 cells, were grown and maintained as monolayers as previously described (Al-Romaiyan et al., 2023a; Marafie et al., 2019) under standard tissue culture conditions. Every 3–4 days the medium was replaced and the when the cells were around 70%–80% confluent they were trypsinized with 0.1% trypsin/0.02% EDTA in preparation for further experimental procedures.

### 2.5 Mouse islet isolation

The pancreata of female BALB/c mice were dissected, and islets were isolated as previously described (Al-Romaiyan et al., 2020a; Al-Romaiyan et al., 2023a) using collagenase IX (Sigma, United States) as a digestion agent. The islets were separated from the exocrine tissues using a histopaque-1077 (Sigma, United States) gradient and sterile-washed three times with RPMI supplemented with 10% fetal bovine serum and 100 U/mL penicillin/0.1 mg/mL streptomycin. The islets were maintained under normal tissue culture conditions (at 37°C and 5% CO_2_) overnight before use for *in vitro* experiments.

### 2.6 Insulin secretion experiments

#### 2.6.1 Static insulin secretion

Static insulin secretion experiments were carried out as described previously (Al-Romaiyan et al., 2020a; Al-Romaiyan et al., 2023a). Mouse islets (3 islets) were pre-incubated in 2 mM glucose physiological buffer supplemented with 0.5 mg/mL BSA and 200 µM CaCl_2_ for 2 h (hrs) at 37°C. After the pre-incubation period, islets were then incubated with 2 mM glucose physiological buffer supplemented with 0.5 mg/mL BSA and 200 µM CaCl_2_ with or without NGR1 (1–100 µM) or 50 µM LY29004 (PI3K inhibitor) for 1 h at 37°C. The supernatant was collected and kept at −20°C until assayed for insulin content.

#### 2.6.2 Perifusion insulin secretion

Mouse islets were perifused using Biorep perifusion system (Biorep, United States), following the manufacturer’s protocol. Fifty mouse islets were placed into perifusion chambers. The chambers were mounted and connected into the system by tubing. The desired buffer for each chamber was selected by an automated valve manifold according to a pre-defined protocol. The perifusion chambers were pre-perifused with 2 mM glucose supplemented with 0.5 mg/mL BSA and 2 mM CaCl_2_ using a high precision peristaltic pump at a flow rate of 0.1 mL/min for 64 min. At 64 min, the islets-containing perifusion chambers were perifused with the agent of interest as detailed in the legend of Figures 2, 5 and the perfusate from each perifusion chambers was collected every 2 min into a multi-well plate. The perfusate was kept at −20°C for assay of insulin content.

### 2.7 RNA extraction, cDNA synthesis and *Ins* mRNA expression measurement

The extraction of RNA, synthesis of cDNA and measurement of *Ins* mRNA expression were performed as previously prescribed (Al-Romaiyan et al., 2020a). Briefly, mouse islets were first pre-incubated with 2 mM glucose for 2 h, before incubation with 2 mM glucose or 100 µM NGR1 or 20 mM glucose for 24 h under usual tissue culture conditions (at 37°C and 5% CO_2_). After this, islets were pooled and washed with ice-chilled phosphate-buffered saline (PBS) and RNA extracted using the Qiagen RNeasy Plus Mini Kit according to manufacturer’s procedure. High-capacity cDNA reverse transcriptase kit with RNase inhibitor (Applied Biosystems, United States) was used to convert 500 ng of RNA to cDNA. RT-PCR was performed using Applied Biosystems™ 7,500 Fast PCR system and LightCycler^®^ FastStart DNA Master PLUS SYBR green I (Roche, United Kingdom) using the following primers: preproinsulin (*Ins*) [Sense: CCA​CCC​AGG​CTT​TTG​TCA; Antisense: TTG​TGG​GTC​CTC​CAC​TTC​A] and actin [Sense: ATG​AAG​TGT​GAC​GTT​GAC​ATC​CGT, Antisense: CCT​AGA​AGC​ATT​TGC​GGT​GCA​CGA​TG] as the housekeeping gene. The Pfaffl method was used for calculating the relative mRNA expression.

### 2.8 Total intra-islet insulin store

Mouse islets (3 islets/Eppendorf tube) were pre-incubated with 2 mM glucose in RPMI for 2 h before incubation with 2 mM glucose, NGR1 (100 µM) or 20 mM glucose for 24 h. Following 24 h, supernatant was collected and kept at −20°C for assay of insulin content. The islets were washed with ice-chilled PBS twice, sonicated with acidified alcohol and kept at −20°C for assay of insulin content.

### 2.9 Measurement of insulin content

Insulin content from supernatant or perfusate or sonicated islets was quantified using the STELLUX^®^ Chemi Rodent Insulin ELISA (ALPCO, United States) as previously described (Al-Romaiyan et al., 2020; Al-Romaiyan et al., 2023a).

### 2.10 Wes™ capillary-based protein electrophoresis

INS-1 832/13 cells were seeded into a 6-well plate as monolayers to a confluency of 70% as previously detailed (Al-Romaiyan et al., 2023a). The cells were then pre-incubated for 2 h at 37°C with physiological buffer supplemented with 2 mM glucose and 2 mM CaCl_2_. After the 2 h pre-incubation period, the cells were incubated with or without 100 µM NGR1 for 5 min. The buffer was aspirated, and cells were washed with cold-iced PBS twice. As previously described (Al-Romaiyan et al., 2023a), cells were lysed, pooled and homogenate was centrifuged for 15 min at 14,000 rpm. The supernatant was collected, transferred to a tube on ice and the protein concentration measured using the Pierce BCA kit (Invitrogen, United States). Proteins were separated using Wes™ capillary (non-gel)-based protein electrophoresis as described previously (Al-Romaiyan et al., 2023a). The primary antibodies (Cell Signaling, United States) used were diluted using an antibody diluent from the kit (anti- β-actin 1:50, total Akt 1:50, phospho-Akt 1:10, total p85 1:50 and phospho-p85 1:10). The data generated were analyzed, calculated, and presented as described in (Al-Romaiyan et al., 2023a).

### 2.11 Cell viability

Cell viability following exposure to NGR1 was evaluated using the CellTiter-Glo^®^ Luminescent Cell Viability Assay (Promega, United States) as previously described (Al-Romaiyan et al., 2020a; Al-Romaiyan et al., 2020b). Briefly, 3 mouse islets were seeded per well in a white 96-well plate and incubated with NGR1 (1–100 µM) at standard tissue culture condition for 24 h. After that CellTiter-Glo^®^ reagent was added to each well and the plate incubated at room temperature for 15 min. The luminescence was read using SpectraMax iD3 (Molecular Devices, United States).

### 2.12 Statistical analysis

Unpaired Student’s t-test, one-way-analysis of variance (ANOVA) followed by Bonferroni’s or Tuckey or Dunnet T-test for multiple comparisons or two-way ANOVA followed by Bonferroni’ or Tuckey or Dunnet T-test for multiple comparisons were used to assess differences between treatment groups as appropriate. Data were represented as mean ± SEM. The differences between treatment groups were considered significant at *p* < 0.05.

## 3 Results

### 3.1 Effect of NGR1 on glucose tolerance *in vivo*

**FIGURE 1:**
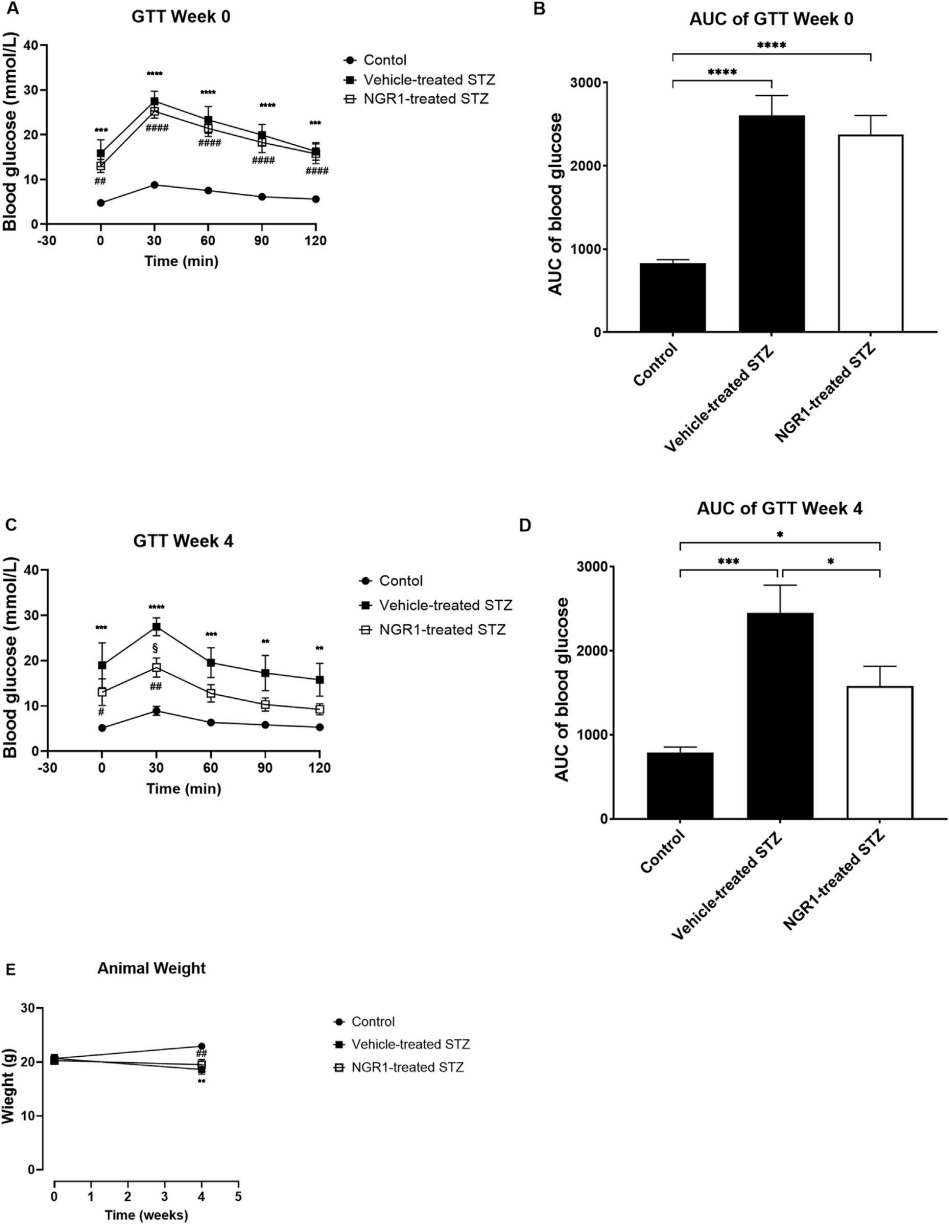
The effect of NGR1 on glucose tolerance test (GTT) from diabetic mice *in vivo*. NGR1 or its vehicle was administered daily by intraperitoneal (IP) injection to diabetic mice for 4 weeks. **(A,C)** Time course of GTT and **(B,D)** area under the curve of the GTT were measured before and after 4 weeks of NGR1 administration. **(E)** Weight of animals. The bars and points represent mean ± SEM. Each experimental group had 5-8 animals per group **(B,D)**: *p < 0.05, ***p < 0.001, ****p < 0.0001 (One-way ANOVA followed by Tukey’s multiple comparisons test and **(A,C,E)**: *p < 0.05, ***p < 0.001, ****p < 0.0001 vehicle-treated STZ vs. control; ##*p* < 0.01, ####*p* < 0.0001 NGR1-treated STZ vs. control; §*p* < 0.05 vehicle-treated STZ vs. NGR1-treated STZ (Two-way ANOVA followed by Tuckey multiple comparisons).

As expected, STZ injection induced diabetes in female BALB/c mice. Both fasting and postprandial blood glucose levels were elevated in STZ diabetic mice as compared to control group. The STZ diabetic mice had the highest blood glucose levels of 27.5 ± 2.2 mmol/L at 30 min post glucose administration, which was significantly higher than the control animals that had 8.7 ± 0.6 mmol/L of blood glucose (*p* < 0.0001; Figure 1A). The AUC of blood glucose was significantly higher in the STZ-treated animals than the non-diabetic control animals (*p* < 0.0001, 2,604 ± 241.0 vs. 827.8 ± 44.49, Figure 1B).

Four weeks’ treatment of NGR1 to diabetic mice significantly improved glucose intolerance as compared to STZ-treated mice without any changes in the weight of the mice (Figures 1C–E) The blood glucose of NGR1-treated diabetic mice was significantly lower than the vehicle-treated diabetic mice (*p* < 0.05, Figures 1C, D). There were no differences in weight between NGR1-treated diabetic mice and the vehicle-treated diabetic mice (*p* > 0.05, Figure 1E).

### 3.2 Acute effect of NGR1 on mouse islets insulin secretion under static insulin secretion setting *in vitro*

**FIGURE 2:**
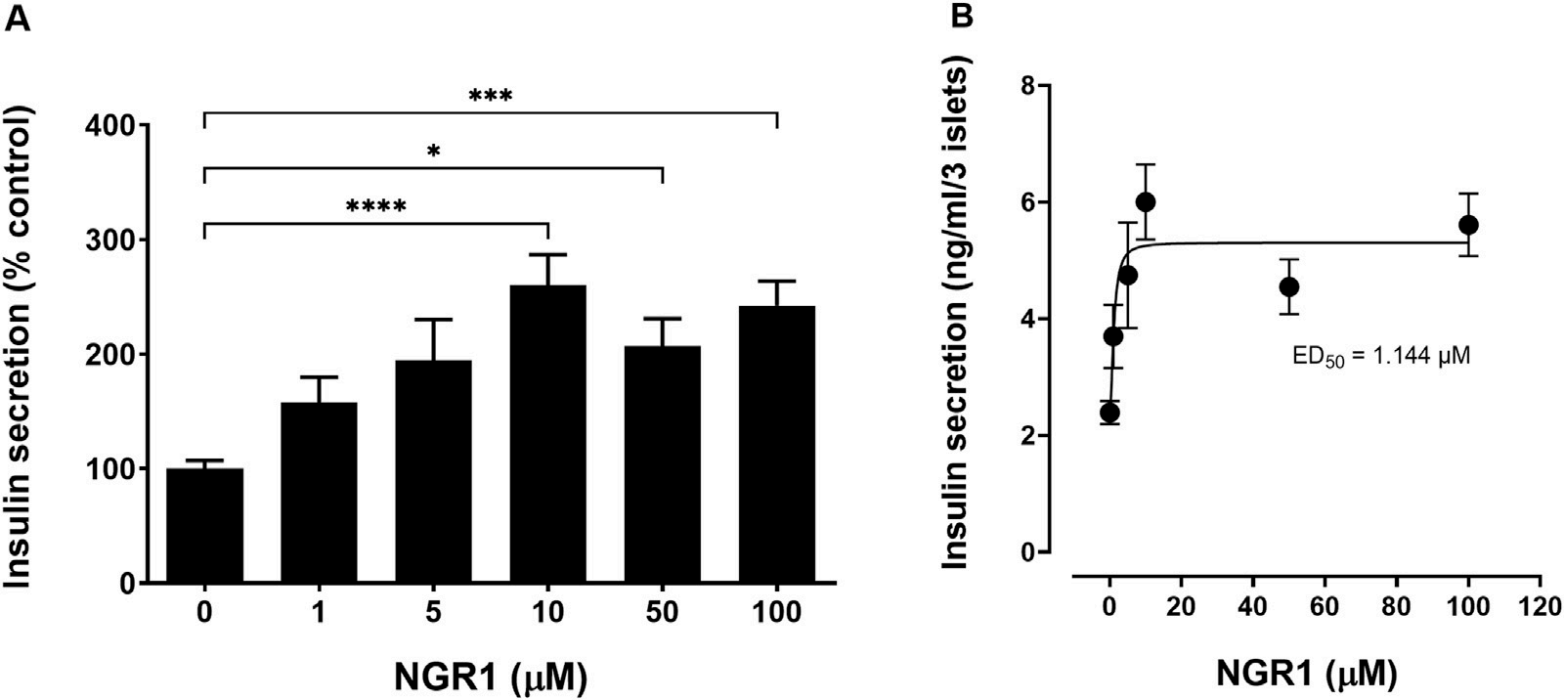
Effect of acute NGR1 incubation on mouse islets insulin secretion. **(A)** Mouse islets were incubated with NGR1 (1–100 µM) at 2 mM glucose for 1 h in a static insulin secretion setting. Insulin concentration was determined from the supernatant. NGR1 caused a concentration-dependent increase in insulin section from mouse islets at 2 mM glucose. The bars represent mean ± SEM for at least three separate experiments. Each experiment had 5–8 replicates per treatment group. *p < 0.05, ***p < 0.001, ****p < 0.0001 vs. 0 µM NGR1 (One way ANOVA followed by Bonferroni’s multiple comparisons test). **(B)** The EC50 of NGR1-induced insulin secretion at 2 mM glucose concentrations. Each point represents mean ± SEM for at least three separate experiments. Each experiment had 5-8 replicates per treatment group.

NGR1 at concentrations of 1–100 µM stimulated mouse islets insulin secretion in a concentration-dependent manner at sub-stimulatory (2 mM) glucose level after a 1 h incubation period (Figures 2A, B). The increase in insulin secretion was significant starting at a concentration of 10 µM (10 µM: 260.3 ± 26.43% vs. 0 µM: 100.1 ± 7.13%, *p* < 0.0001) and was maintained even when increasing NGR1 concentration up to 100 µM.

### 3.3 Acute effect of NGR1 on mouse islets insulin secretion under insulin secretion perifusion setting *in vitro*

**FIGURE 3:**
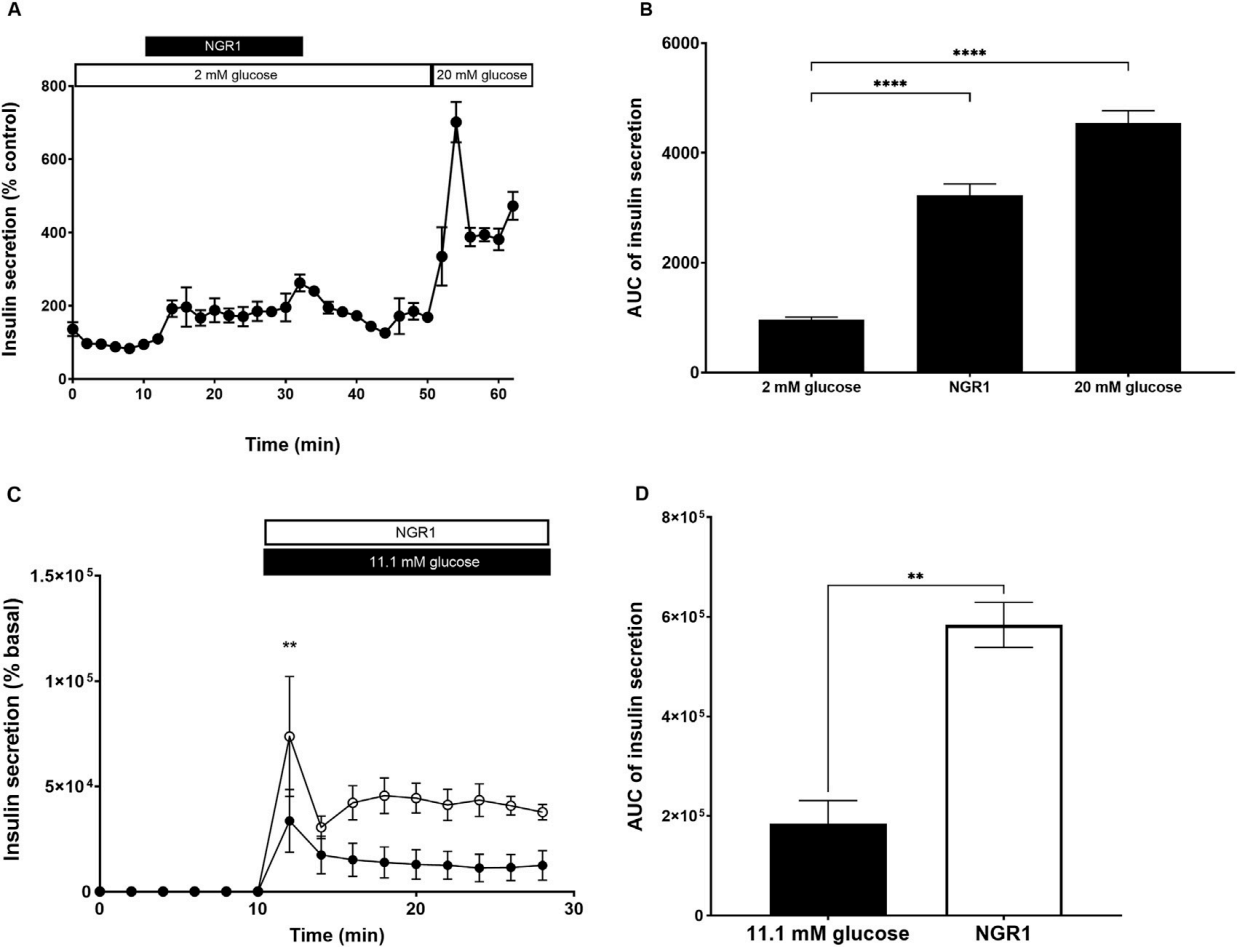
Effect of NGR1 on the rate and pattern of mouse islets insulin secretion. Fifty islets were loaded into perifusion chambers and pre-perifused with 2 mM glucose physiological buffer for 64 min and the perfusate was discarded. **(A, B)** The islets were perifused with 2 mM glucose or 2 mM glucose + 100 µM NGR1 or 20 mM glucose in a timely fashion (0–62 min) and perfusate was collected every 2 min at a flow rate of 0.1 mL/min. The insulin content was determined from the perfusate. The data was plotted as a treatment-time graph **(A)** or as area under the curve of each treatment slot graph **(B)**. NGR1 caused a rapid and sustained increase in insulin secretion. The points and bars represent mean ± SEM, n = 3. Each replicate represents 50 islets from a pool of 3 mice. Data are representative of two separate experiments. ****p < 0.0001 vs. 2 mM glucose (One-way ANOVA followed by Dunnett’s multiple comparisons test). **(C, D)** The islets were perifused with 2 mM glucose (0–10 min) before being challenged with 11.1 mM glucose (black circle) or 11.1 mM glucose + 100 µM NGR (white circle) (10–30 min) and perfusate was collected every 2 min at a flow rate of 0.1 mL/min. The insulin content was determined from the perfusate. The data was plotted as a treatment-time graph **(C)** or as area under the curve of each treatment slot graph **(D)**. The points and bars represent mean ± SEM, n = 3. Each replicate represents 50 islets from a pool of 6 mice. **p < 0.01 vs. 11.1 mM glucose (unpaired Student’s t-test or Two-way ANOVA followed by Bonferroni’s multiple comparisons test).

The effect of NGR1 on the pattern and rate of insulin secretion was assessed using perifusion system which allows for a more sensitive assessment of changes in the rate of insulin secretion. At basal insulin secretion, addition of NGR1 (for 10–30 min) significantly initiated insulin secretion from perifused mouse islets. This stimulation of insulin secretion was maintained throughout NGR1 perifusion period (AUC of 0–10 min 2 mM glucose: 959.7 ± 47.92 vs. AUC of 11–30 min NGR1: 3,222 ± 211.7, *p* < 0.0001). Upon withdrawal and washing of NGR1 (31–50 min), the insulin secretion did not return to basal levels instead a new basal insulin level was created (AUC of 0–10 min 2 mM glucose: 959.7 ± 47.92 vs. AUC of 31–50 min 2 mM glucose: 3,269 ± 155.4, *p* < 0.0001; Figures 3A, B). Perifusing mouse islets with 20 mM glucose (51–62 min) stimulated a two-phased insulin secretion. The first phase of the glucose-induced insulin secretion was rapid, high in amplitude and lasted for about 4 min while the second phase was lower in amplitude and lasted throughout the rest of the 20 mM glucose perifusion period (Figure 3A).

Perifusing mouse islets with 11.1 mM glucose, a more physiological postprandial glucose concentration, stimulated a two-phased insulin secretion which was augmented with the addition of NGR1 (Figures 3C, D). NGR1 enhanced both the first and second phase of glucose-induced insulin secretion. NGR1 at 2 min following glucose challenge caused a significant increase in insulin secretion over 11.1 mM glucose (NGR1: 73,695 ± 28,509 vs. 33,648 ± 14,869, *p* < 0.01).

### 3.4 Chronic effect of NGR1 on insulin secretion and synthesis and cell viability *in vitro*

**FIGURE 4:**
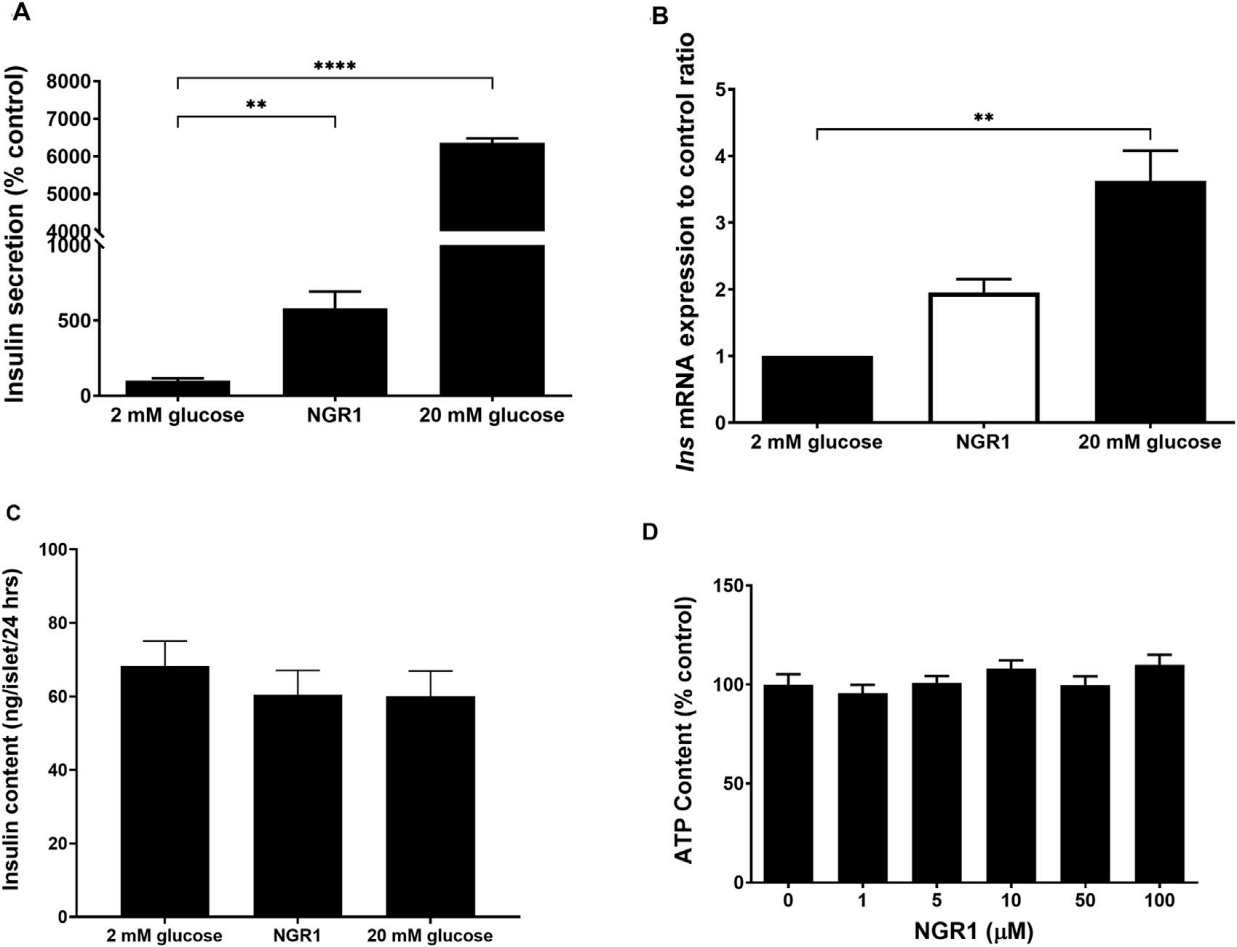
Effect of chronic NGR1 treatment on insulin secretion, preproinsulin mRNA expression, total intra-islet insulin content and mouse islets cell viability. Mouse islets were incubated with NGR1 (100 µM) for 24 h, **(A)** Insulin concentration was determined from the supernatant and **(B)** *Ins* mRNA expression was determined. **(C)** Mouse islets were sonicated in acidified alcohol and total intra-islet insulin content was determined. The bars represent mean ± SEM for at least three separate experiments. Each experiment had 3 replicates per treatment group for mRNA expression experiments and 6-8 replicates per treatment group for insulin secretion and intra-islet insulin content experiments. **p < 0.01, ****p < 0.0001 vs. 2 mM glucose (One-way ANOVA followed by Dunnett’s multiple comparisons test). **(D)** Mouse islets (3 islets/well) were seeded in a white 96-well plate and treated with NGR1 (1–100 µM) for 24 h. ATP content as an indicator of cell viability was determined by CellTiter-Glo^®^ Luminescent Cell Viability Assay (Promega, United States). The bars represent mean ± SEM of three separate experiments. Each experiment had 6-8 replicates per treatment group. *p* > 0.05 (One-way ANOVA followed by Dunnett’s multiple comparisons test).

Chronic incubation of mouse islets with NGR1 stimulated insulin secretion over a period of 24 h (Figure 4A) as compared to 2 mM glucose (581.3% ± 110.8% vs. 100 ± 16.14, *p* < 0.01). 20 mM glucose also chronically increased insulin secretion following 24 h exposure. These increases in insulin levels in 20 mM glucose-treated mouse islets were accompanied with significant increases in the mRNA expression of *Ins* (3.63 ± 0.79-fold change, *p* < 0.01). However, increases in insulin levels in NGR1-treated mouse islets were not accompanied with significant increases in the mRNA expression of *Ins* (1.95 ± 0.19-fold change, *p* > 0.05, Figure 4B). Their total intra-islet insulin store did not change after either treatment (Figure 4C).

Exposing mouse islet to different concentrations of NGR1 (1–100 µM) for chronic (24 h) incubation time was not associated with reduction in cellular ATP content (Figure 4D). This indicated that the viability of mouse islets was maintained and preserved following chronic NGR1 exposure.

### 3.5 The signaling pathway mediating the NGR1-induced insulin secretion *in vitro*

**FIGURE 5:**
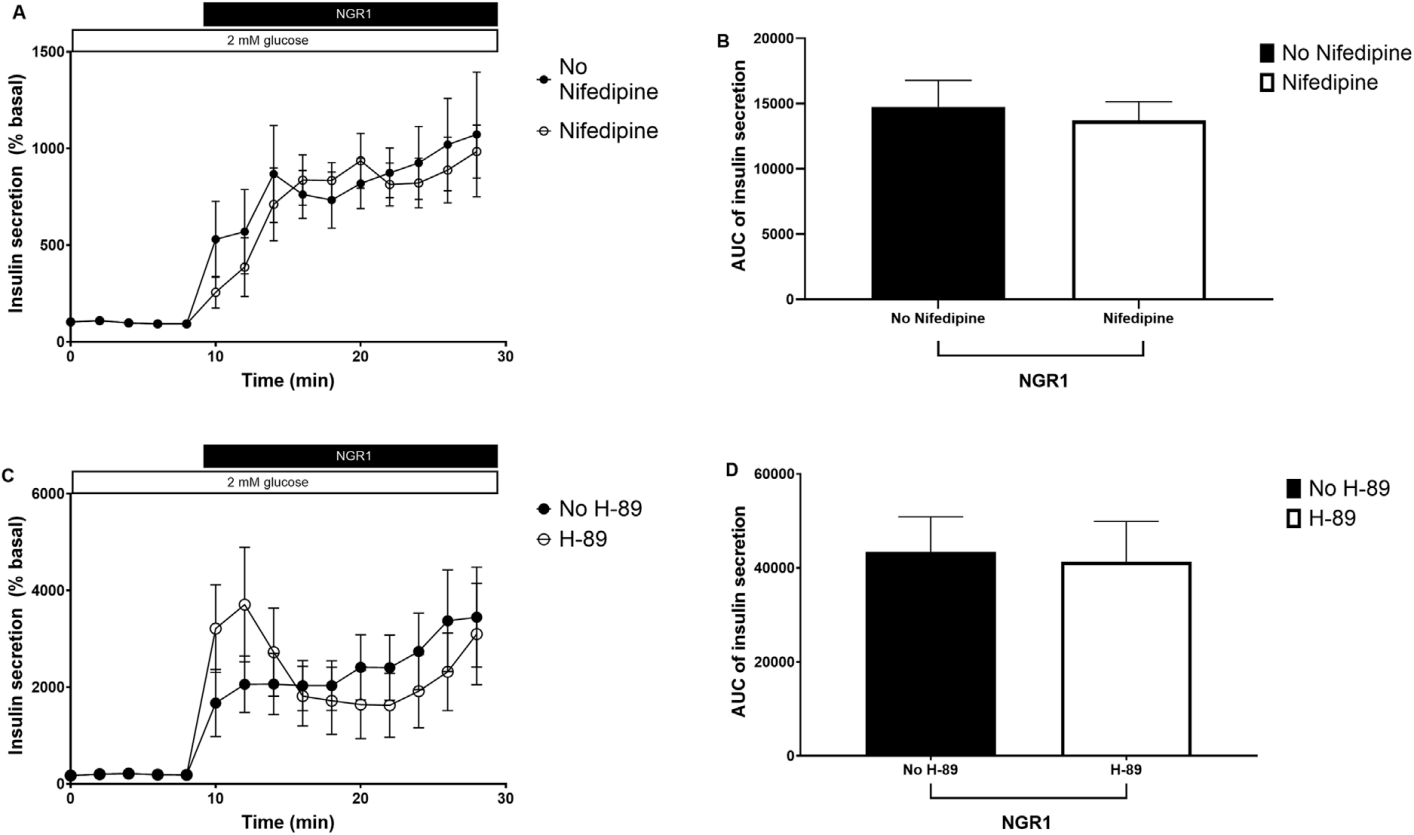
The effect of NGR1 on voltage gated calcium channels (VGCC) and protein kinase A (PKA) pathway. Fifty islets were loaded into perifusion chambers and pre-perifused with 2 mM glucose physiological buffer for 64 min and the perfusate was discarded. The islets were then perifused with 100 µM NGR1 in the presence or absence of nifedipine **(A, B)** and H-89 **(C, D)** and perfusate was collected every 2 min at a flow rate of 0.1 mL/min. The insulin content was determined from the perfusate. The data was plotted as a treatment-time graph **(A, C)** or as area under the curve of each treatment slot graph **(B, D)**. NGR1 caused a rapid and sustained increase in insulin secretion that is not affected by blocking Ca^2+^ influx through VGCC or inhibiting PKA activation. The points and bars represent mean ± SEM, n = 6. Each replicate represents 50 islets from a pool of 6 mice. *p* > 0.05 (unpaired Student’s t-test or Two-way ANOVA followed by Bonferroni’s multiple comparisons test).

Incubation mouse islets with 100 µM NGR1 in a perifusion setting increased insulin secretion. The presence of 10 µM nifedipine, a voltage gated calcium channels (VGCC) blocker, or 10 μM H-89, a PKA inhibitor did not inhibit NGR1-induced insulin secretion (Figures 5A–D), even though both nifedipine and H-89 blocked glucose-stimulated insulin secretion (GSIS) and forskolin augmentation of GSIS, respectively (Supplementary Figure S1).

**FIGURE 6:**
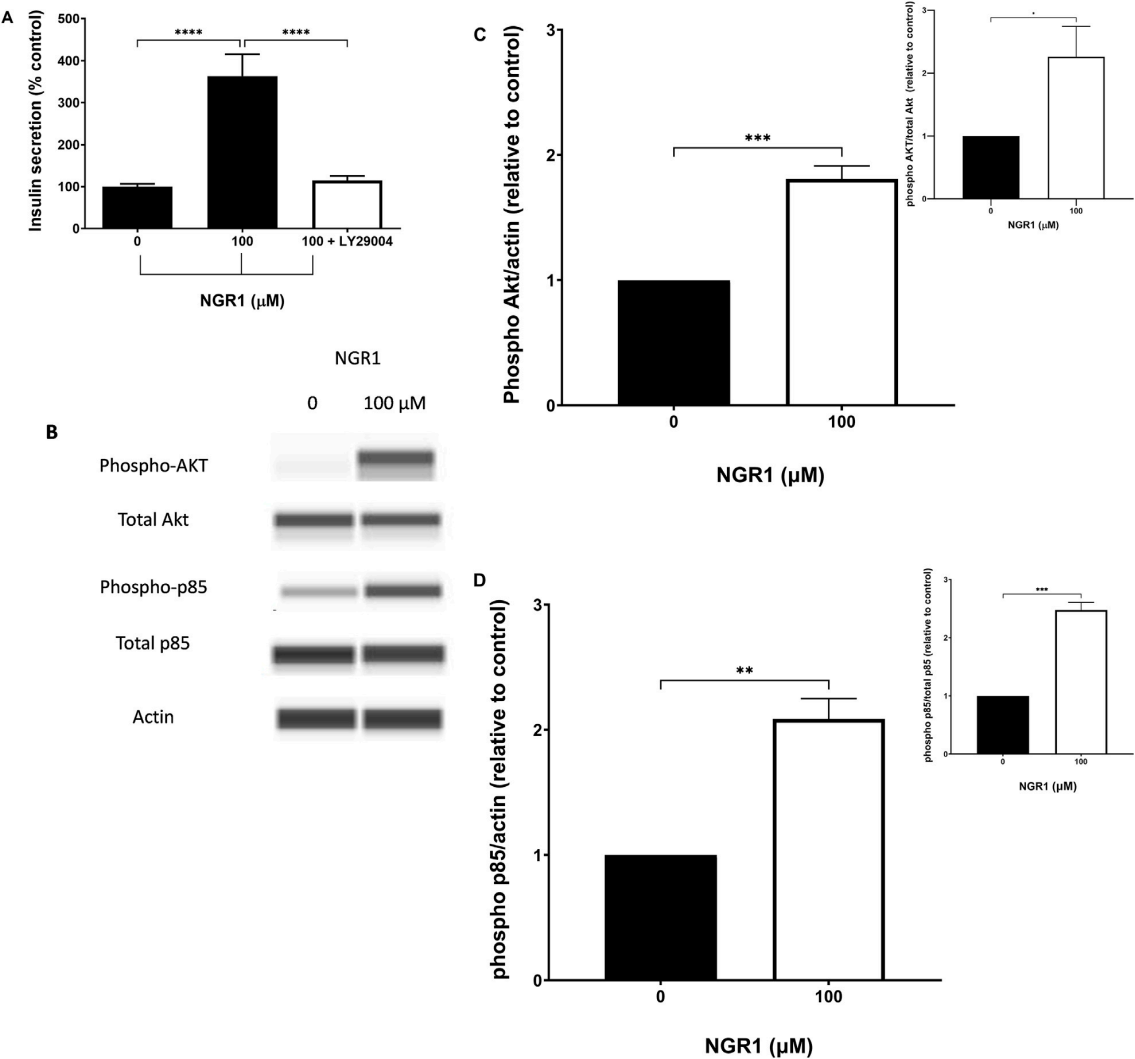
The effect of NGR1 on PI3K/Akt pathway. **(A)** Mouse islets (3 islets/tube) were pre-incubated with 2 mM glucose for 2 h before being incubated with 100 µM NGR1 in the presence or absence of 50 µM LY29004 (a PI3K inhibitor) for 1 h at 37°C. Insulin content was determined from the supernatant. Data are mean ± SEM of at least three separate experiments. Each experiment had 5–12 replicates per treatment group. ****p < 0.0001 (One-way ANOVA followed by Bonferroni’s multiple comparisons test). **(B–D)** INS-1 832/13 cells were incubated for 5 min with NGR1 (100 µM) for the measurement of Akt and p85 phosphorylation. Cells were lysed and 3 µg of protein was used. Expression of phosphorylated and total protein of interest was detected by Wes™ capillary-based protein electrophoresis and normalized to β-actin (and total protein; inset) and expressed relative to control. The bars represent mean ± SEM of 3–4 separate experiments. Each experiment had 3 replicates per treatment group. *p ˂ 0.05, **p < 0.01, ***p < 0.001 (Unpaired t-test).

Incubating mouse islets with 100 µM NGR1 in a static secretion setting caused a dramatic increase in insulin secretion as expected (363.2% ± 52.16% vs. 100 ± 6.7, p < <0.0001). However, The NGR1-induced insulin secretion was completely abolished in the presence of 50 µM LY29004, a PI3K inhibitor (Figure 6A).

In addition, exposure to 100 µM NGR1 for 5 min elevated the phosphorylation level of p85, which is the regulatory subunit of PI3K, and Akt in INS-1 832/13 cells (Figures 6B–D).

## 4 Discussion

Treatment of T2DM using botanical drugs is widely accepted by the public and extensively investigated by researchers. Many plants have been reported to be efficacious in the treatment of T2DM. PNG metabolites have also been recently reported to possess antidiabetic properties (Chen et al., 2008; Uzayisenga et al., 2014; Yang et al., 2010). NGR1, a major metabolite of PNG roots, has been suggested to have anti-diabetic properties. Therefore, using our custom-design screening method (Al-Romaiyan et al., 2023b), we investigated the therapeutic potential of NGR1 as an antidiabetic agent by creating a “screening portfolio” for NGR1. The current study shows that NGR1 was able to lower blood glucose *in vivo* in animal model of diabetes. NGR1 was shown for the first time to stimulate insulin secretion directly from mouse islets at basal and postprandial glucose concentrations *in vitro*. The NGR1 stimulation of insulin secretion was dependent on activation of PI3K/Akt pathway.

Our interest was to study the molecular mechanism effects of NGR1’s antidiabetic effects. Since there is discrepancy in the literature regarding the *in vivo* studies, a single dose exploratory study was done to check on which side of the argument NGR1 lies and to reduce to the minimum the number of animals used. The single dose showed that NGR1 has antidiabetic effects and thus we focused on studying the molecular mechanism *in vitro*.

To resolve the discrepancy in the results reporting the antidiabetic effect of NGR1 *in vivo*, the glucose-lowering effect of NGR1 was investigated in a female STZ-mouse model, a model of diabetes, *in vivo*. Our data demonstrated that 4 weeks administration of NGR1 to female STZ mice significantly improved glycemia in these animals consistent with other studies that reported an improvement in glucose tolerance in a male db/db mouse model of diabetes following 10 weeks (Zhai et al., 2018) and 20 weeks (Zhang et al., 2018) of NGR administration. These studies attributed the beneficial effect of NGR1 to the amelioration of insulin resistance rather than improvement in β-cell function. However, several other studies have shown that there were no significant changes in fasting blood glucose following NGR1 administration (Yang et al., 2010; Zhang et al., 2019). Another study reported a reduction in fasting blood glucose level in a male STZ rat model of diabetes following 16 weeks of NGR1 administration (Huang et al., 2016). These studies were focused on assessing the effect of NGR1 on diabetic complications such as diabetic encephalopathy and nephropathy rather than on the function of islets and insulin secretion. In addition, these studies utilized male models of diabetes and none of the published studies so far tested the effect of NGR1 in a STZ female BALB/c mouse model of diabetes as we did in this study. Our data showed that NGR1 did not change the weight of the mice following 4-week of treatment suggesting that NGR1 effect on the levels of glucose is not due to weight loss.

On-going *in vivo* studies are currently being conducted in our laboratory to test the effect of NGR1 on blood glucose level using more than one dose, compare the activity of NGR1 to known antidiabetic agents such as sulphonylureas or GLP-1 agonists, identify the underlying mechanism(s) and pathway(s) by which NGR1 might alleviate glucose intolerance. Our preliminary data have shown that NGR1 has a tendency to increase plasma insulin levels *in vivo* following chronic intake (STZ group: 0.5303 ± 0.2362 ng/mL; STZ + NGR1 group: 1.586 ± 0.3118 ng/mL, n = 3-5, *p* = 0.05, unpaired t-test). It is noteworthy to mention that our data is the first to report an increase in plasma insulin levels following NGR1 administration in an insulin-deficient animal model of diabetes.

Since our data showed that NGR1 has a glucose lowering effect *in vivo*, we focused on identifying the molecular mechanism of NGR1 *in vitro*. To investigate whether NGR1 has a direct activity on β-cell islets of Langerhans, static insulin secretions were performed. NGR1 directly stimulated insulin secretion, in a concentration-dependent manner, from isolated mouse islets at basal glucose concentrations under static insulin secretion setting where measurements of accumulated insulin levels occurred over a single time point (60 min in our experiments). These data indicate that the glucose-lowering effect of NGR1 seen in the *in vivo* study is most likely due to direct insulin stimulation from β-cells islets of Langerhans. Therefore, NGR1 could behave similarly to the currently available insulin secretagogue agents such as sulphonylureas and GLP-1 agonists.

It is noteworthy to mention that the basal glucose concentration was maintained at 2 mM glucose in all our experiments. This glucose concentration is sub-stimulatory and was used to eliminate the contribution of glucose to the secretory response (Al-Romaiyan et al., 2010). The increase in insulin levels induced by NGR1 was significant at NGR1 concentrations of ≥10 µM with an EC50 of 1.14 µM. The ability of NGR1 to initiate insulin secretion at a glucose sub-stimulatory concentration of 2 mM is suggestive of a mechanism of action that is independent of nutrient metabolism.

Perifusion experiments with isolated mouse islets were used to evaluate the kinetic profile of NGR1 on insulin secretion to further elucidate the pattern, reversibility and rate of insulin secretion induced by NGR1. The rate and pattern of insulin secretion can be determined during perifusion experiments since insulin output can be measured from sequential samples over a given frequency (every 2 min in our experiment setting) (Al-Romaiyan et al., 2020a; Al-Romaiyan et al., 2023b). NGR1 caused a rapid and sustained increase in insulin secretion at basal glucose concentrations following perifusing of mouse islets with NGR1. The NGR1-induced insulin secretion was reversible, although a new basal for insulin levels was created following withdrawal of NGR1. This could suggest that NGR1 may sensitize the insulin secretory machinery making β-cells more responsive to glucose. Further stimulation of insulin secretion upon the exposure to a stimulatory glucose concentration (20 mM) following NGR1 treatment, confirmed that exposure to NGR1 was not associated with β-cell damage. This is because the after exposure to NGR1, cells exposed to glucose were subsequently able to metabolize it and trigger membrane depolarization.

NGR1 also potentiated glucose-induced insulin secretion following exposing mouse islets to 11.1 mM glucose, a physiological postprandial glucose concentration. In fact, NGR1 augmented both phases of glucose-stimulated insulin secretion indicating that NGR1 also acts by potentiating glucose-induced insulin secretion to reduce postprandial hyperglycemia similar to GLP-1 agonists, well-known antidiabetic class of drugs. The ability of NGR1 to potentiate phase 1 and 2 of glucose-stimulated insulin secretion could indicate that NGR1 may target early and late rise of glucose levels in the blood following a carbohydrate rich meal.

Some plant metabolites may stimulate insulin secretion through unregulated insulin release via increasing membrane permeability and thus may have deleterious effect on cell viability (Al-Romaiyan et al., 2020a; Al-Romaiyan et al., 2020b; Liu et al., 2009). To ascertain that NGR1 stimulated insulin secretion without affecting membrane integrity, viability of cells following exposure to NGR1 was measured using ATP viability test. Chronic exposure of mouse islets to NGR1 was not associated with any reduction in cell viability. This demonstrates that insulin secretion induced by NGR1 occurred through a regulated exocytosis of insulin from β-cells without any deleterious effect on membrane integrity and cell viability.

Preservation of β-cell stores through enhancement of insulin biosynthesis is an important characteristic in an insulin secretagogue agent (Al-Romaiyan et al., 2023b). To investigate whether the NGR1-induced insulin secretion is accompanied by increases in insulin synthesis, mRNA expression of preproinsulin (*Ins*) was measured and compared to 20 mM glucose, a powerful inducer of *Ins* expression. In our experiment, 20 mM glucose chronically stimulated insulin secretion and increased preproinsulin (*Ins*) gene expression while maintaining β-cell stores, which is consistent with published studies that reported a stimulation of *Ins* gene expression by glucose in β-cell lines (da Silva Xavier et al., 2000; Rafiq et al., 2000) and primary islets (Evans-Molina et al., 2007). Similarly, NGR1 increased insulin secretion in chronically treated mouse islets following 24 h incubation and maintained total intra-islet β-cell insulin stores. This may suggest that NGR1 may initiate insulin secretion through a mechanism that involves activation of one or more signaling pathways linked to β-cell stimulus-secretion coupling.

Insulin exocytosis and release from β-cells is primarily triggered by calcium entry through VGCC following glucose metabolism and phosphorylation and ATP-sensitive potassium channels closure (Campbell and Newgard, 2021; Jones and Persaud, 1998). Insulin secretion can be largely modulated by activation of second messengers such as PI3K and cAMP and the subsequent stimulation of mediators such as Akt and, PKA (Jones and Persaud, 1998). To investigate the reliance of the insulin stimulatory effect of NGR1 on these signaling pathways, the effect of known pharmacological inhibitors on NGR1-induced insulin secretion and the effect of NGR1 on protein expression of these signaling molecules were measured. Data from our insulin secretion experiments have shown that NGR1 initiated mouse islets insulin secretion independently of calcium entry through VGCC and PKA activation as evidenced by maintaining of NGR1-induced insulin secretion despite blockade of VGCC by nifedipine and inhibition of PKA by the pharmacological inhibitor H-89. On the contrary, NGR1-induced insulin release was completely abolished by the pharmacological inhibitor of PI3K, LY29004. In addition, data from our Western blotting experiments demonstrated that NGR1 increased phosphorylated p85, the regulatory subunit of PI3K, and Akt, the downstream effector of PI3K, indicating that NGR1 acts through activation of PI3K/Akt pathway to increase insulin secretion. The dependency of NGR1-induced insulin secretion on PI3K/Akt pathway in mouse islets is consistent with other published studies that reported the involvement of this pathway in NGR1 action on various biological systems (Lei et al., 2022; Li et al., 2022a; Meng et al., 2014; Sun et al., 2013; Tu et al., 2018; Wang et al., 2021).

In conclusion, our study is the first to report that NGR1 has a direct insulin secretagogue activity from β-cells islets of Langerhans. Based on our custom-designed screening method, NGR1 has fulfilled most of the criteria for an effective antidiabetic agent that works through targeting β-cell function. It directly potentiates glucose-induced insulin secretion through activating PI3K/Akt pathway, a known pathway in stimulus-secretion coupling, maintains membrane integrity and β-cell viability, sustains intra-islet β-cell store, and improves hyperglycemia *in vivo* in an animal model of diabetes. NGR1 has therapeutic potential as an antidiabetic agent that can be used as an adjunctive therapy for T2DM.

## Data Availability

The raw data supporting the conclusions of this article will be made available by the authors, without undue reservation.
