# Supplementary figures and images for: Notoginsenoside R1, a metabolite from Panax notoginseng (Burkill) F.H.Chen, stimulates insulin secretion through activation of phosphatidylinositol 3-kinase (PI3K)/Akt pathway
Source: Front Pharmacol. 2024 Sep 27;15:1478917. doi: 10.3389/fphar.2024.1478917 (PMC11466869; doi:10.3389/fphar.2024.1478917)

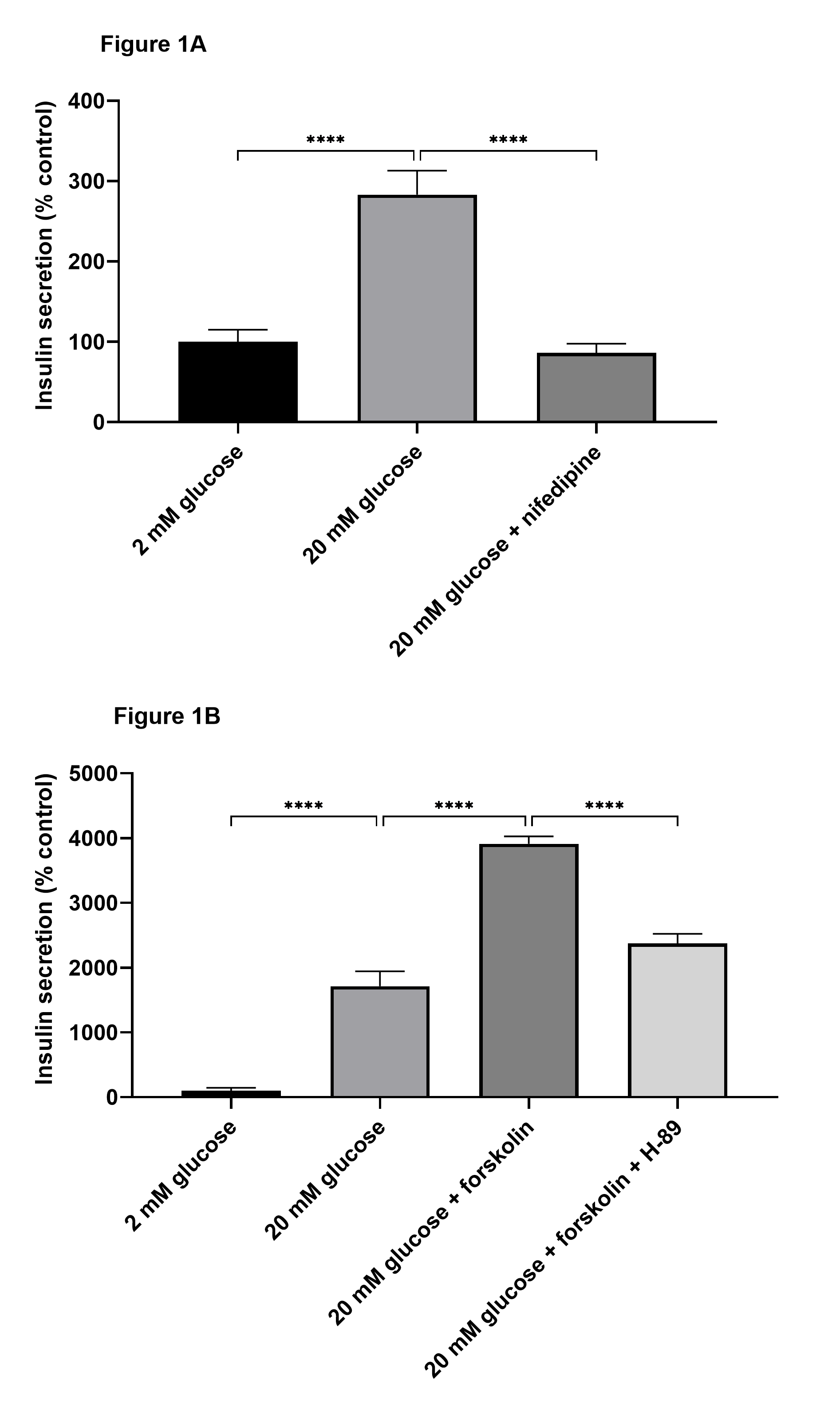

Supplement: Supplementary file 1 [file Image1.TIF]
